# Transgender female sex workers’ HIV knowledge, experienced stigma, and condom use in the Dominican Republic

**DOI:** 10.1371/journal.pone.0186457

**Published:** 2017-11-02

**Authors:** Henna Budhwani, Kristine R. Hearld, Julia Hasbun, Rebecca Charow, Santo Rosario, Louise Tillotson, Elaine McGlaughlin, John Waters

**Affiliations:** 1 Department of Health Care Organization and Policy, University of Alabama at Birmingham, Birmingham, AL, United States of America; 2 Department of Health Services Administration, University of Alabama at Birmingham, Birmingham, AL, United States of America; 3 Centro de Orientación e Investigación Integral (COIN), Calle Arzobispo Meriño, Santo Domingo, Dominican Republic; 4 Caribbean Vulnerable Communities Coalition (CVC), Kingston, Jamaica; 5 United Nations Population Fund (UNFPA), Tegucigalpa, Honduras; Massachusetts General Hospital, UNITED STATES

## Abstract

**Introduction:**

Not only do transgender female sex workers have some of the highest rates of sexually transmitted infections (STI), human immunodeficiency virus (HIV), and experienced stigma, they also have higher likelihood of early sexual debut and some of the lowest levels of educational attainment compared to other stigmatized populations. Some of the most common interventions designed to reduce transmission of HIV and STIs seek to educate high-risk groups on sexual health and encourage condom use across all partner types; however, reaching stigmatized populations, particularly those in resource-limited settings, is particularly challenging. Considering the importance of condom use in stopping the spread of HIV, the aim of this study was two-fold; first to characterize this hard-to-reach population of transgender female sex workers in the Dominican Republic, and second, to assess associations between their HIV knowledge, experienced stigma, and condom use across three partner types.

**Methods:**

We analyzed self-reported data from the Questionnaire for Transgender Sex Workers (N = 78). Respondents were interviewed at their workplaces. Univariate and bivariate analyses were employed. Fisher Chi-square tests assessed differences in HIV knowledge and experienced stigma by condom use across partner types.

**Results:**

HIV knowledge was alarmingly low, condom use varied across partner type, and the respondents in our sample had high levels of experienced stigma. Average age of first sexual experience was 13.12 years with a youngest age reported of 7. Dominican Republic statutory rape laws indicate 18 years is the age of consent; thus, many of these transgender women’s first sexual encounters would be considered forcible (rape) and constitute a prosecutable crime. On average, respondents reported 8.45 sexual partners in the prior month, with a maximum of 49 partners. Approximately two thirds of respondents used a condom the last time they had sex with a regular partner. This was considerably lower than condom use reported with coercive partners (92.96%) and clients (91.78%). Bivariate analyses revealed two trends: experienced stigma was associated with lower rates of condom use, and lower HIV knowledge was associated with lower rates of condom use. The former provides additional evidence that experienced stigma may become internalized, affecting individual-level behaviors—lowering self-confidence and resilience—making it more difficult to negotiate condom use due to lack of self-efficacy and desire to show trust in one’s partner. The latter supports public health research that suggests gaps in HIV knowledge persist and are pronounced in highly stigmatized populations.

**Discussion:**

The vulnerabilities experienced by transgender persons, particularly in environments that vehemently adhere to conservative ideologies related to sex and gender, are significant and harm this population. These vulnerabilities could potentially be addressed through critically examining of impact of policies that indirectly promote or allow victimization of transgender citizens and subsequently diminish the effectiveness of public health and educational interventions. By taking action through the revocation of such laws, the Dominican Republic has the opportunity to improve overall population health, to protect some of its most stigmatized citizens, and to become the flag bearer of enhanced human rights in the Caribbean and Latin America.

## Introduction

Not only do transgender female sex workers have some of the highest rates of sexually transmitted infections (STI), human immunodeficiency virus (HIV), exposures to violence, experienced stigma, and experiences with discrimination, they also have higher likelihood of early sexual debut and some of the lowest levels of educational attainment compared to other stigmatized and high-risk for HIV populations.[1–2] Globally, transgender women have an HIV prevalence ranging from 17.7% to 21.6%[3] with transgender female sex workers being even more at-risk with an estimated HIV prevalence of 27.3%.[4] In the Dominican Republic, transgender female sex workers’ HIV prevalence is about 17%, lower than in other middle-income nations but still devastatingly high.[5] Some of the most common evidence-based interventions designed to reduce HIV/STI transmission seek to educate high-risk groups on sexual health and encourage condom use across all partner types;[1] however, reaching stigmatized populations, particularly those who fear experiencing stigma through physical abuse and emotional victimization is challenging.[6–7] Additionally, improving HIV knowledge in these populations may not be enough to increase condom use, since stigmatized populations are more likely to engage in risk-behaviors (e.g. not using condoms) as coping mechanisms in response to internalized and experienced stigma, such discrimination and violence.[1,8–9] Considering the importance of condom use in stopping the transmission of HIV and STIs, the aim of this study was two-fold; first to characterize this hard-to-reach population, and second, to assess associations between HIV knowledge, experienced stigma, and condom use across three partner types in a sample of transgender female sex workers in the Dominican Republic.

Few studies exist on the experiences of transgender female sex workers in the Dominican Republic; consequently, little is known about HIV/STI prevalence and transmission. What we do know is that STI prevalence among transgender female sex workers in Latin America is high; the Pan American Health Organization (PAHO) conducted a regional study and found 42–50% of transgender female sex workers had syphilis, 42% had hepatitis B, and over 97% tested positive for human papillomavirus.[10] Regardless of geographic location, sex work is associated with engagement in high risk behaviors and an increased likelihood of acquiring and transmitting HIV[1–2] and STIs.[11–12] In fact, studies comparing HIV rates in transgender female sex workers to cisgender sex workers found transgender female sex workers had higher rates of HIV.[6–7,12–14] Since societal norms, values, and constructs determine how biological sex and gender are viewed, understood, and accepted, transgender individuals may encounter social stigma, institutional discrimination, and violence due to their gender presentation which is incongruent with their biological sex.[15] Societal-level stigma not only increases likelihood of being victimized but also increases the likelihood that transgender persons will engage in high risk-behaviors.[12,16–19] Additional motivations for engagement in high risk-behaviors include seeking social acceptance, financial necessity, and engagement in risk-behaviors as a coping mechanism, all of which impact condom use.[19–20] Transgender female sex workers’ cumulative disadvantage lowers resilience and self-confidence, resulting in transgender female sex workers being less able to negotiate condom use, putting them at greater risk for HIV and STIs.[7,10] Research on transgender women and gender an sexual minorities suggest that fear of losing intimacy, perception of minimal health risk, seeking of validation from partner, perceived negative attitudes towards condoms from partners, substance use, and misinformation regarding HIV transmission are all possible explanations for low condom use.[21–24]

Considering this knowledge gap, we examined associations between experienced stigma, HIV knowledge, and condom use across three partner types—clients, coercive partners, and trusted partners. This study is particularly novel, because we disaggregated behavior by partner type, which has only occurred in a rare few studies with transgender populations.[25] In terms of understanding differences in partner types: clients (also called “customers” by respondents) are those who pay for sexual acts. Coercive partners (also referred to as “casual partners” by respondents) are those who are in a position of authority over the transgender female sex worker, such as a landlord or a police officer. Coercive partners do not pay money or provide goods for sex; they receive sexual favors from transgender female sex workers for simply being powerful.[26] Trusted partners are committed partners such as a boyfriend, girlfriend, significant other, etc.[7,22–24] Recent studies exploring transgender female sex workers’ behavior found that respondents were most likely to not use a condom with their trusted partner(s) as compared to other types of partners.[22–24] Complementary studies suggest this lack of condom use with their trusted partners is due to a desire for closeness and intimacy with this partner type,[11] but not necessarily with coercive partners and clients.[27–28] Research with cisgender sex workers found similar and consistent patterns of condom non-use with trusted partners.[11,29–31] Therefore, research on motivations and barriers to condom use and condom non-use would benefit from 1) parsing behaviors by partner types, 2) considering the psychology of relationships and intimacy, and 3) examining how seemingly unrelated social exposures and experiences with stigma may be associated with condom use and non-use.[32]

## Materials and methods

We analyzed data from the Questionnaire for Transgender Sex Workers in the Dominican Republic (N = 78). These data were collected in Santo Domingo, Dominican Republic and Santiago, Dominican Republic by the Caribbean Vulnerable Communities Coalition (CVC) and the Centro de Orientación e Investigación Integral (COIN). Participant inclusion criteria were: 1) Having exchanged goods or money for sexual acts in the past year, 2) Being at least eighteen years old, and 3) Self-identifying as a transgender woman. The study period ran from December 2013 to April 2014 with data collection occurring in February 2014. Two trained research assistants interviewed transgender female sex workers at their workplaces, which included street zones, sex work businesses, and hotels with short-term (hourly) rental policies. Snowball sampling was employed. Surveys were conducted in Spanish.

Ethical approval to conduct this study provided by El Consejo Nacional de Bioética en Salud, (CONABIOS) in the Dominican Republic; approval included the collection of informed consent verbally rather than in writing. Verbal consent is a modality we often use in participatory action research when working with populations with low literacy or reflecting cultural acceptability.[33–34] We have found verbal consent, when designed and implemented thoughtfully with attention to cultural appropriateness, leads to thorough comprehension of the study and of voluntary participation as opposed to securing written consent, which may cause psychosocial discomfort when the respondent cannot read or write.[35–36] The verbal consent process involved three steps: 1) Provision of a comprehensive verbal description about the study and participant’s rights in layman’s terms, 2) An explanation of what was being asked of the potential participant, and 3) Requesting and obtaining potential participants verbal consent that she was freely participating. Verbal consent with each transgender female sex worker participant was witnessed and audio recorded. The witness was always a senior co-Investigator. Ethical approval for secondary analysis of these de-identified data was obtained from University of Alabama at Birmingham’s (UAB) Institutional Review Board (#N150803004).

### Measures

The primary outcomes for this study were condom use with trusted partners, condom use with coercive partners, and condom use with clients. Respondents were asked: “The last time you had sex with a ([type] partner) did you use a condom?” with answer choices of no and yes (no = 0; yes = 1). Explanations of each partner type were provided to the respondent (e.g. trusted meaning spouse, boyfriend, or girlfriend and coercive meaning landlord, police officer, pimp, etc.). Personal characteristics were assessed with three variables: age, education, and main source of income. Age is a demographic indicator, while education and income are socioeconomic indicators. Age was measured as a continuous variable. Education was measured categorically. Age and education have been shown to affect condom use; higher levels of education are linked to higher rates of condom use, and younger age is associated with fewer negative perceptions around condom use.[37,38] Three sources of income were included: sex work, family support, and other jobs. Sexual history was assessed with two continuous variables: age of first sexual experience with penetration and number of sexual partners in the last month.

HIV knowledge was assessed with nine dichotomous measures. Respondents were asked if they believe HIV was transmitted through: 1) Mosquito bites, 2) Sharing of food, 3) Pregnancy to unborn child, 4) Breastfeeding, 5) Sex without a condom once, 6) Anal sex, 7) Public bathroom use, and 8) Oral sex. The ninth question—measuring HIV knowledge—asked if the respondent believed that most people with HIV would die soon. These measures were all binary coded with answer choices of no and yes (no = 0; yes = 1). Experienced stigma was assessed with three dichotomous questions on: ease of accessing medical care, being calling names, and experience with physical violence. Specifically, respondents were asked: “Forgetting the money, is it easy or difficult to obtain medical care for your needs?” (easy = 0; difficult = 1). Experience with violence was measured by asking, “Have you ever been a victim of physical violence at least once by a client [customer]?” (no = 0; yes = 1), and each transgender female sex worker respondent was asked if people at work called her “nasty” (Spanish translation) names (no = 0; yes = 1).

### Statistical analyses

Univariate statistics described characteristics of this sample of transgender female sex workers. Bivariate analyses were also employed. Fisher Chi-square tests assessed differences in HIV knowledge and experienced stigma by condom use across three partner types—trusted, coercive, and client. All analyses were conducted using SAS version 9.3.

## Results

Univariate results are in Table 1. Average age of our sample was 23 years; transgender female sex workers’ ages ranged from 18 to 39. No respondents were aged 40 or older at the time of the study. Almost 17% (16.67%) had an elementary level education, and 35.90% had some university education. Three fourths reported sex work as their main source of income (76.92%); other respondents reported sex work as a secondary or tertiary source of income. The average age of first sexual experience with penetration was 13.12 years (ranged from 7 to 20 years) which is significantly lower than the minimum age of consent (18 years) in the Dominican Republic.[39] On average, respondents reported 8.45 sexual partners in the prior month, with a maximum of 49 partners. Approximately two thirds of transgender female sex workers used a condom the last time they had sex with a trusted partner (67.76%). This was considerably lower than condom use reported with coercive partners and clients; 92.96% of transgender female sex workers reported using a condom the last time they had sex with a coercive partner, and 91.78% reported using a condom the last time they had sex with a client.

**Table 1:** Univariate statistics of transgender female sex worker respondents.

	N/% (Yes)
***Demographics***	
Age (Mean/Standard Deviation)	23.01 / 4.76
Education	
Elementary	13 / 16.67%
Some Secondary	29 / 37.18%
Secondary Completed	29 / 37.18%
College or University	28 / 35.90%
Main source of money	
Sex Work	60 / 76.92%
Family Support	14 / 17.95%
Other Job	4 / 5.13%
Age of first sexual experience with penetration (Mean/Standard Deviation)	13.12 / 3.23
Number of sexual partners in the last month (Mean/Standard Deviation)	8.45 / 7.90
***Condom Use***	
The last time you had sex with a regular partner you used a condom?	42 / 67.76%
The last time you had sex with a coercive partner you used a condom?	66 / 92.96%
The last time you had sex with a client you used a condom?	67 / 91.78%
***HIV Knowledge***	
Can a person get HIV from mosquito bites?	55 / 70.61%
Can a person get HIV by sharing food with someone who is infected?	57 / 73.08%
Can an infected pregnant woman transmit the HIV virus to her unborn child?	59 / 75.64%
Can a woman transmit the HIV virus to their newborn son through breastfeeding?	55 / 70.51%
Can a person get HIV if he / she has sex without a condom only once?	63 / 80.77%
Is HIV only transmitted by having anal sex?	35 / 44.87%
Do you think that you can get HIV by having oral sex?	62 / 79.49%
Do you think that most people with HIV will die from it soon?	62 / 79.49%
***Experienced Stigma***	
When you are out of money, is it easy or difficult to obtain medical care for your needs? Difficult	57 / 73.08%
Do people at work call you nasty names?	33 / 42.31%
Have you ever been a victim of physical violence by your clients?	33 / 42.31%

In terms of HIV knowledge, over two thirds of transgender female sex workers believed that a person could contract HIV from mosquito bites (70.61%, false statement); 73.08% thought HIV could be transmitted by sharing food (false statement); 75.64% thought that a pregnant woman could transmit HIV to her unborn child (true statement), and 70.51% believed a woman could transmit HIV through breastfeeding (true statement). Over 80% understood that HIV could be transmitted through sex without a condom, even if only once (80.77%); only 55.13% believed there were multiple methods to transmit or contract HIV. Less than half believed that HIV was only transmitted by anal sex (44.87%). Almost 80% knew that HIV could be transmittable by oral sex (79.49%), and 79.49% believed that those with HIV would die from it soon.

Experienced stigma was high in our sample. Over 40% (42.31%) of transgender female sex workers reported people calling them nasty (translated from Spanish) names at work; the same percentage (42.31%) reported being physically victimized by clients. Almost three quarters (73.08%) of transgender female sex workers reported difficulty obtaining medical care. This number is particularly startling, because the Dominican Republic has a three-tiered health care system, which theoretically guarantees access to free, socialized health care for the poor.[40]

Bivariate associations are reported in Table 2. Over 98% of transgender female sex workers who answered yes to the question, “Can a person get HIV if he/she has sex without a condom only once?” used a condom the last time they had sex with a coercive partner (98.28%), compared to 69.23% of transgender female sex workers who answered no (χ2 = 13.69, p<0.05). Over 96% of transgender female sex workers who thought a person could get HIV by sharing food with someone infected used a condom the last time they had sex with a client (96.23%), compared to 80.00% of transgender female sex workers who answered no (χ2 = 5.07, p<0.05). Likewise, 96.55% of respondents who believed a person can get HIV if he/she has sex without a condom only once used a condom during their last sexual experience with a client, compared to 73.33% of respondents who answered no (χ2 = 8.52, p<0.05).

**Table 2:** Bivariate association between condom use during last sexual encounter across three partner types.

	Condom Use with Regular Partner N = 42			Condom Use with Coercive Partner N = 66			Condom Use with Client N = 67		
	Yes	No	χ2	Yes	No	χ2	Yes	No	χ2
***HIV Knowledge***									
Can a person HIV from mosquito bites?	31 (72.09%)	11 (57.89%)	χ2 = 1.22 p = 0.38	49 (96.08%)	17 (85.00%)	χ2 = 2.69 p = 0.13	46 (92.00%)	21 (91.30%)	χ2 = 0.01 p = 0.99
Can a person get HIV by sharing food with someone who is infected?	32 (71.11%)	10 (58.82%)	χ2 = 0.85 p = 0.38	47 (94.00%)	19 (90.48%)	χ2 = 0.28 p = 0.63	51 (96.23%)	16 (80.00%)	**χ2 = 5.07 p<0.05**
Can an infected pregnant woman transmit the HIV virus to her unborn child?	30 (63.83%)	12 (80.00%)	χ2 = 1.36 p = 0.35	50 (92.39%)	15 (94.12%)	χ2 = 0.05 p = 0.83	50 (89.29%)	17 (100.00%)	χ2 = 1.98 p = 0.33
Can a woman transmit the HIV virus to their newborn son through breastfeeding?	28 (62.22%)	14 (82.35%)	χ2 = 2.29 p = 0.22	47 (95.92%)	19 (86.36%)	χ2 = 2.11 p = 0.17	46 (92.00%)	21 (91.30%)	χ2 = 0.01 p = 0.99
Can a person get HIV if he / she has sex without a condom only once?	22 (66.00%)	9 (75.00%)	χ2 = 0.35 p = 0.74	57 (98.28%)	9 (69.23%)	**χ2 = 13.69 p<0.05**	56 (96.55%)	11 (73.33%)	**χ2 = 8.52 p<0.05**
Is HIV only transmitted by having anal sex?	19 (67.86%)	23 (67.65%)	χ2 = 0.00 p = 0.99	35 (94.59%)	31 (91.18%)	χ2 = 0.32 p = 0.67	31 (93.94%)	36 (90.00%)	χ2 = 370.00 p = 0.68
Do you think you can get HIV by using a public bathroom?	27 (69.23%)	15 (65.22%)	χ2 = 0.11 p = 0.78	43 (93.48%)	23 (92.00%)	χ2 = 0.05 p = 0.82	42 (91.30%)	25 (92.59%)	χ2 = 0.04 p = 0.99
Do you think that you can get HIV by having oral sex?	31 (63.27%)	11 (85.62%)	χ2 = 2.14 p = 0.19	53 (92.98%)	13 (92.86%)	χ2 = 0.00 p = 0.99	53 (92.98%)	14 (87.50%)	χ2 = 0.50 p = 0.61
Do you think that most people with HIV will die from it soon?	33 (64.71%)	9 (81.82%)	χ2 = 1.21 p = 0.48	51 (91.07%)	15 (100.00%)	χ2 = 1.44 p = 0.58	52 (89.66%)	15 (100.00%)	χ2 = 1.69 p = 0.33
***Experienced Stigma***									
Out of money, is it easy or difficult to obtain medical care for your needs?	34 (77.27%)	8 (44.44%)	**χ2 = 6.30 p<0.05**	54 (98.18)	12 (75.00%)	**χ2 = 10.17 p<0.01**	50 (96.15%)	17 (80.95%)	**χ2 = 4.58 p<0.05**
Do people at work call you nasty names?	14 (66.67%)	28 (68.29%)	χ2 = 0.02 p = 0.99	26 (83.87%)	40 (100.00%)	**χ2 = 6.94 p<0.05**	26 (81.25%)	41 (100.00%)	**χ2 = 8.38 p<0.01**
Have you ever been a victim of physical violence by your clients?	12 (50.00%)	28 (80.00%)	**χ2 = 5.87 p<0.05**	26 (83.87%)	37 (100.00%)	**χ2 = 6.44 p<0.05**	27 (81.82%)	37 (100.00%)	**χ2 = 7.36 p<0.01**

Only 44.44% of transgender female sex workers who answered that it was difficult to obtain medical care for health care needs used a condom during their last sexual experience with a trusted partner, compared to 77.27% of respondents who reported no difficulty (χ2 = 6.30, p<0.05). Similarly, 75.00% transgender female sex workers who answered that it was difficult to obtain medical care used a condom during their last sexual experience with a coercive partner, compared to 98.18% of the transgender female sex workers who answered it was easy to obtain health care (χ2 = 10.17, p<0.01); 80.95% transgender female sex workers who answered that it was difficult to obtain medical care used a condom during their last sexual experience with a client, compared to 96.15% of the transgender female sex workers who answered it was easy to obtain care (χ2 = 4.58, p<0.05). Approximately 84% (83.87%) of transgender female sex workers who reported being called derogatory names at work used a condom the last time they had sex with a coercive partner, compared to 100.00% of the transgender female sex workers who had not experienced verbally discrimination (χ2 = 6.94, p<0.05). Likewise, 81.25% of transgender female sex workers reported being called derogatory names at work used a condom the last time they had sex with a client, compared to 100.00% of the transgender female sex workers who had not experienced stigma verbally (χ2 = 8.38, p<0.01). There was a significant association between condom use and exposure to physical violence across all three partner types. Fifty percent of transgender female sex workers who reported having been a victim of violence used a condom in their last sexual experience with a trusted partner, compared to 80.00% who had not been physically victimized (χ2 = 5.87, p<0.05). Similarly, 83.87% of transgender female sex workers who had experienced physical violence used a condom the last time they had sex with a coercive partner, compared with 100.00% of transgender female sex workers who had not experienced violence (χ2 = 6.44, p<0.05). Finally, 81.82% of those who had experienced physical violence used a condom the last time they had sex with a client, compared to 100.00% of those who had not (χ2 = 7.36, p<0.01).

## Discussion

We found that HIV knowledge was low, condom use varied across partner type, and that the transgender female sex workers in our sample had high levels of experienced stigma, specifically verbal discrimination, physical victimization, and difficulty accessing health care. Average age of transgender female sex workers’ first sexual experience was 13.12 years with a youngest age reported of 7. Dominican Republic statutory rape laws indicate that 18 years is the age of consent; thus, many of these women’s first sexual encounters would be deemed forcible and constitute a crime against a minor.[38,39] Bivariate analyses revealed two significant trends: experienced stigma was associated with lower rates of condom use, and lower HIV knowledge was associated with lower rates of condom use. The former adds support to the idea that experienced stigma may become internalized, lowering self-confidence and resilience, making it more difficult or less desirable to negotiate condom use.[11,21] The latter adds to the body of public health research and practice which suggests that gaps in HIV knowledge persist, and these knowledge gaps are more pronounced in stigmatized populations.

Unfortunately, there are few evidence-based HIV educational interventions for transgender female sex workers in the Dominican Republic or across the Spanish speaking Caribbean. One study which engaged cisgender female sex workers in Puerto Plata and Santo Domingo found that interventions combining community solidarity with government policy actions were effective in increasing condom use with trusted partners and reducing STI prevalence.[41] However, the lack of public support for and acceptance of transgender populations and sex work in the Dominican Republic could pose a major obstacle to ensuring governmental action.[42–43] More scientific research into transgender female sex workers’ health behaviors, motivations, and HIV/STI knowledge levels is necessary to better understand the profile of this population; further investigations could be informative in aiding the development of educational and behavioral interventions, and may assist domestic and international human rights advocates to accurately represent the needs of transgender individuals.

Limitations of this study should be considered when applying or extending findings. First, bias is to be expected in self-reported survey data, especially when the topic is perceived to be controversial or the study population is highly stigmatized. For example, the high rate of “always” responses to the frequency of condom use questions could be attributed to the unbounded recall time—“the last time you had sex…,” but could also reflect a social desirability bias. Some of the study measures lacked specificity, reducing extensibility of our findings. For instance, although we assessed difficulty in accessing medical care regardless of availability of funds, we did not have information about challenges accessing medical care specifically due to being a transgender women or a sex worker. Having the latter would have better defined specific barriers associated with health care access for this population. Additionally, interpersonal characteristics vary even within partner types (e.g. coercive partners), in that a police officer may assert a greater degree of influence on a transgender female sex worker than a landlord. Both were classified as coercive partners; thus, greater granularity in subsequent studies may be particularly informative in better understanding the role of power and authority. Our sample size was small; more comprehensive data collection could provide deeper, generalizable insights. Finally, causality cannot be inferred from cross-sectional data.

## Conclusions

In many parts of the world, transgender women are marginalized, victimized, and stigmatized. In the Dominican Republic, discrimination against transgender women is systemic.[3] Although progress has been made to recognize transgender rights in some high-income countries, these steps forward have not translated into universal support or human right protections of transgender people around the world.[44] Transgender people (and transgender female sex workers) continue to experience stigma that robs them of respect, economic opportunities, and dignity.[44–49] These experiences have detrimental effects on the health and wellbeing of transgender people.[44–49] Experienced stigma, discrimination and physical violence may reduce the likelihood of condom use, increase engagement in highly risky sexual behaviors, and in turn, increase rates of HIV and STIs in transgender populations.[21,44–45,48–49] The vulnerabilities experienced by transgender persons, especially transgender persons in resource-constrained settings such as in the Dominican Republic, could be partially addressed through understanding of impact of policies that indirectly promote or allow harm to transgender citizens and subsequently diminish the effectiveness of public health and education interventions that are funded both by domestic and global agencies that seek to contain the HIV epidemic.[44] By taking these actions through the revocation of such laws and making these societally relevant commitments, the Dominican Republic has the opportunity to improve overall population health, to protect some of its most stigmatized citizens, and to become the flag bearer of enhanced human rights in the Caribbean and Latin America.

## Supporting information

S1 File Restricted base data. (PDF)
