# Supplementary material for: Transgender female sex workers’ HIV knowledge, experienced stigma, and condom use in the Dominican Republic
Source: PLoS One. 2017 Nov 2;12(11):e0186457. doi: 10.1371/journal.pone.0186457 (PMC5667872; doi:10.1371/journal.pone.0186457)
Supplement: S1 File — (PDF) [file pone.0186457.s001.pdf]

B	C	P1	P2	P3	P4	P5	P6	P7	P8	P9	P10	P11	P12	P13	P14	P15	P16	P17	P18	P19	P20	P21	P22
1	1	12	1	1	0	1	4	1	0	10	14	0	9	5	0	M	M	M	M	M	M	1	1
1	1	12	1	1	0	0	3	2	0	13	4	0	2	2	0	M	M	M	M	M	M	1	1
1	1	13	1	1	3	0	3	3	0	15	5	0	3	2	0	M	M	M	M	M	M	1	1
1	1	13	1	1	0	0	16	M	0	14	10	0	10	0	0	M	M	M	M	M	M	1	1
1	1	12	2	1	1	1	16	M	0	13	3	0	2	1	0	M	M	M	M	M	M	1	1
1	1	9	1	0	0	0	134	5	0	15	25	0	25	0	0	M	M	M	M	M	M	1	0
1	1	12	1	0	4	0	16	M	0	7	7	0	3	4	0	M	M	M	M	M	M	1	1
1	1	9	1	1	2	1	4	1	0	14	7	0	6	1	0	M	M	M	M	M	M	1	1
1	1	12	1	1	2	1	16	M	0	15	3	8	1	3	0	M	M	M	M	M	M	1	0
1	1	7	2	1	2	1	16	M	0	20	1	0	20	0	0	M	M	M	M	M	M	0	M
1	1	12	1	1	2	1	16	M	0	18	9	0	6	3	0	M	M	M	M	M	M	1	1
1	1	12	2	0	4	1	16	M	0	16	5	0	2	3	0	M	M	M	M	M	M	1	1
1	1	12	1	1	3	1	16	M	0	14	2	0	2	0	0	M	M	M	M	M	M	1	1
1	1	12	1	1	2	1	16	M	0	17	14	0	14	0	0	M	M	M	M	M	M	1	1
1	1	5	1	1	2	1	16	M	0	10	14	0	12	2	0	M	M	M	M	M	M	1	1
1	1	10	1	1	0	1	16	M	0	10	14	0	9	5	0	M	M	M	M	M	M	1	1
1	1	9	1	1	0	0	16	M	0	13	4	0	2	2	0	M	M	M	M	M	M	1	1
1	1	9	1	0	0	0	134	5	0	15	18	0	18	0	0	3	0	M	6	3	1	1	0
1	1	12	1	1	2	1	16	M	0	15	3	8	1	3	0	3	0	M	6	3	1	1	0
1	1	7	2	1	2	1	16	M	0	20	1	0	20	0	0	M	M	M	M	M	M	0	M
1	1	13	1	1	0	2	16	M	0	12	4	1	2	2	1	1	M	1	M	1	0	1	1
1	1	8	1	1	1	1	16	M	0	11	4	2	1	1	1	4	0	M	6	0	0	1	1
1	1	12	1	1	1	1	3	3	0	14	10	0	6	4	1	4	0	M	6	0	0	1	0
1	1	7	1	1	1	1	16	M	0	9	20	4	20	5	1	3	0	M	6	0	1	1	1
1	1	13	1	1	2	1	16	M	0	11	2	1	0	1	1	3	0	M	6	3	0	1	1
1	1	3	1	0	4	0	16	M	0	11	3	1	0	2	1	2	0	M	1	2	1	1	1
1	1	12	1	1	0	2	16	M	0	15	3	1	2	0	1	4	0	M	6	0	0	0	M
1	1	5	1	1	0	2	16	M	0	8	1	3	1	0	1	2	0	M	6	3	0	1	1
1	1	10	3	0	2	2	16	M	0	12	9	4	5	0	1	3	0	M	6	0	0	1	1
1	1	9	1	0	2	0	16	M	0	12	16	3	15	0	1	3	0	M	6	3	0	1	1
1	1	12	2	1	2	1	16	M	0	9	13	1	8	4	1	3	0	M	1	3	0	1	1
1	1	12	1	0	2	2	16	M	0	15	16	5	12	4	1	3	0	1	6	3	0	1	1
1	1	11	1	1	2	1	16	M	0	11	1	3	0	1	1	4	0	M	6	0	1	1	0
1	1	4	1	0	4	0	16	M	0	8	11	1	5	1	1	3	0	M	6	3	0	1	1
1	1	12	1	1	2	1	16	M	0	14	4	1	1	2	1	3	0	M	88	3	1	1	1
1	1	7	1	1	1	1	16	M	0	9	20	4	20	5	1	3	0	M	6	0	1	1	1
1	1	10	1	1	2	1	16	M	0	11	2	1	0	1	1	3	0	M	6	3	0	1	1
1	1	10	1	1	0	2	16	M	0	15	3	1	2	0	1	4	0	M	6	0	0	0	M
1	1	5	1	1	0	2	16	M	0	8	1	3	1	0	1	2	0	M	6	3	0	1	1
1	1	12	1	0	2	2	16	M	0	15	16	5	12	4	1	3	0	1	6	3	0	1	1
1	1	10	1	1	2	1	16	M	0	11	1	3	0	1	1	4	0	M	6	0	1	1	0
1	1	12	1	1	2	1	16	M	0	14	4	1	1	2	1	3	0	M	88	3	1	1	1
1	1	10	1	1	0	1	16	M	0	10	4	16	3	1	1	2	1	1	M	2	0	1	1
1	1	12	3	1	1	1	16	M	0	10	1	3	0	1	1	1	1	1	M	1	1	1	1
1	1	7	1	1	2	1	16	M	0	12	2	2	1	0	1	1	1	1	M	1	0	1	1
1	1	10	1	1	0	1	16	M	0	16	6	2	5	1	1	1	1	1	M	M	1	1	1

1	1	13	2	1	3	1	16	M	0	13	5	1	3	2	1	2	1	1	M	2	0	1	1
1	1	10	5	1	0	0	16	M	0	7	8	1	0	8	1	1	1	1	M	M	0	1	1
1	1	5	1	1	2	1	1	1	0	9	1	1	15	0	1	1	1	1	M	1	1	0	M
1	1	13	1	1	0	2	16	M	0	13	5	M	1	0	1	1	1	1	M	0	0	0	M
1	1	10	1	1	2	1	4	5	0	13	1	3	0	0	1	1	1	1	M	1	1	1	1
1	1	9	1	1	0	2	16	M	0	9	0	1	0	0	1	2	1	1	M	2	0	1	1
1	1	12	2	1	0	2	16	M	0	14	3	1	0	2	1	1	1	1	M	1	0	1	1
1	1	8	1	1	4	0	16	M	0	15	3	2	1	0	1	1	1	1	M	1	0	1	1
1	1	2	1	0	4	0	16	M	0	8	15	2	14	0	1	1	1	1	M	1	0	0	M
1	1	12	1	0	0	0	16	M	0	12	3	1	2	0	1	1	1	1	M	1	0	1	1
1	1	12	1	1	1	1	4	1	0	17	11	1	10	0	1	1	1	1	M	1	0	1	1
1	1	13	2	1	1	1	16	M	0	13	3	1	0	3	1	1	1	1	M	M	0	1	1
1	1	4	5	1	3	1	4	1	0	17	1	1	0	0	1	1	1	1	M	1	0	0	M
1	1	11	1	0	2	0	16	M	0	16	7	2	5	0	1	1	1	3	M	1	1	1	1
1	1	12	1	0	2	1	16	M	0	16	10	1	7	2	1	1	1	1	M	1	0	1	1
1	1	7	8	0	1	2	16	M	0	16	9	1	5	3	1	1	1	1	M	1	0	1	1
1	1	10	1	1	2	1	16	M	0	16	2	2	1	0	1	1	1	3	M	1	0	1	1
1	1	7	1	1	2	1	16	M	0	10	23	1	20	3	1	1	1	3	M	M	0	1	1
1	1	12	1	1	2	1	16	M	0	20	6	1	2	4	1	1	1	1	M	1	1	1	1
1	1	12	1	1	3	2	16	M	0	17	9	2	6	3	1	3	1	1	M	0	1	1	1
1	1	12	1	1	2	0	16	M	0	16	16	2	5	10	1	1	1	1	M	1	1	1	1
1	1	12	1	1	2	1	16	M	0	9	24	1	20	3	1	1	1	1	M	1	0	1	1
1	1	12	2	1	2	0	16	M	0	13	15	0	5	10	1	1	1	3	M	1	1	1	1
1	1	11	1	0	3	1	16	M	0	15	7	1	6	10	1	1	1	1	M	1	0	1	1
1	1	11	1	1	2	0	16	M	0	12	49	2	28	20	1	1	1	3	M	1	1	1	1
1	1	9	1	1	3	1	16	M	0	12	3	4	1	1	1	1	1	1	M	1	0	1	1
1	1	9	1	1	2	1	16	M	0	10	45	4	24	20	1	1	1	3	M	1	0	1	1
1	1	8	1	1	3	2	16	M	0	13	9	2	6	3	1	1	1	1	M	M	0	1	1
1	1	12	2	1	3	1	16	M	0	18	14	5	3	10	1	1	1	3	M	1	1	1	1
1	1	12	2	1	2	1	16	M	0	16	5	2	3	2	1	1	1	1	M	1	0	1	1
1	1	10	1	1	0	1	16	M	0	16	6	2	5	1	1	1	1	1	M	M	1	1	1
1	1	12	2	1	3	1	16	M	0	13	5	1	3	2	1	2	1	1	M	2	0	1	1
1	1	9	5	1	0	0	16	M	0	7	8	1	0	8	1	1	1	1	M	M	0	1	1
1	1	2	1	0	4	0	16	M	0	8	15	2	14	0	1	1	1	1	M	1	0	0	M
1	1	10	1	0	0	0	16	M	0	12	3	1	2	0	1	1	1	1	M	1	0	1	1
1	1	10	1	1	1	1	4	1	0	17	11	1	10	0	1	1	1	1	M	1	0	1	1
1	1	13	2	1	1	1	16	M	0	13	3	1	0	3	1	1	1	1	M	M	0	1	1
1	1	10	1	1	2	1	16	M	0	16	2	2	1	0	1	1	1	3	M	1	0	1	1
1	1	7	1	1	2	1	16	M	0	10	23	1	20	3	1	1	1	3	M	M	0	1	1
1	1	8	1	1	2	1	16	M	0	20	6	1	2	4	1	1	1	1	M	1	1	1	1
1	1	10	1	1	3	2	16	M	0	17	9	2	6	3	1	3	1	1	M	0	1	1	1
1	1	10	1	1	2	0	16	M	0	16	16	2	5	10	1	1	1	1	M	1	1	1	1
1	1	11	1	1	2	1	16	M	0	9	24	1	20	3	1	1	1	1	M	1	0	1	1
1	1	12	2	1	2	0	16	M	0	13	15	0	5	10	1	1	1	3	M	1	1	1	1

P23	P24	P25	P26	P27	P28	P29	P30	P31	P32	P33	P34	P35	P36	P37	P38	P39	P40	P40b	P41	P42
1	M	1	1	15	1	1	9	5	1	1	M	1	1	4	1	1	1	4	1	1
1	M	1	0	20	1	1	2	2	1	1	M	1	0	2	1	1	1	2	1	1
1	M	1	1	18	1	4	3	2	1	1	M	1	1	4	1	1	1	2	1	1
1	M	1	0	23	1	1	10	0	1	1	M	1	0	4	1	1	1	M	1	1
1	M	1	1	14	1	4	2	1	1	1	M	1	1	4	1	1	1	1	1	1
M	4	4	0	17	1	1	25	0	0	M	4	3	1	2	0	1	0	M	1	1
1	M	1	0	23	1	1	3	4	1	1	M	1	0	4	1	0	0	M	1	1
1	M	1	1	18	1	1	6	1	1	1	M	1	1	4	1	0	0	M	1	1
M	1	3	0	16	1	1	6	9	1	1	M	2	0	4	1	1	1	4	1	1
M	M	M	M	14	1	1	20	0	1	3	M	1	0	4	1	0	0	M	3	1
3	M	1	1	18	1	1	6	3	1	1	M	1	1	4	1	1	0	M	3	1
3	M	1	0	20	1	1	2	3	1	1	M	1	0	4	1	1	1	4	1	1
1	M	1	1	14	1	1	2	0	1	1	M	1	0	4	1	0	1	4	1	1
1	M	1	1	17	1	1	14	0	1	1	M	1	1	4	1	1	1	4	1	1
3	M	1	0	12	1	1	12	2	1	1	M	1	0	4	1	1	0	M	1	1
1	M	1	1	15	1	1	9	5	1	1	M	1	1	4	1	1	1	4	1	1
1	M	1	0	20	1	1	2	2	1	1	M	1	0	2	1	1	1	2	1	1
M	4	4	0	17	1	1	18	0	1	1	M	3	1	2	0	1	0	M	1	1
M	1	3	0	16	1	1	6	9	1	1	M	2	0	4	1	1	1	4	1	1
M	M	M	M	14	1	1	20	0	1	3	M	1	0	4	1	0	0	M	3	1
1	M	1	0	16	1	1	2	2	1	1	M	1	1	4	1	1	1	2	2	1
1	M	1	1	16	1	1	1	3	1	1	M	1	1	2	0	1	1	2	1	1
M	1	2	1	17	1	1	6	4	1	1	M	1	1	4	1	1	1	2	1	1
3	M	1	0	9	1	14	20	4	1	1	M	1	0	4	1	1	1	2	1	1
3	M	1	1	20	1	4	0	2	1	1	M	M	0	4	0	1	1	1	1	1
1	M	1	1	11	1	4	0	3	1	1	M	1	0	4	1	1	1	M	1	2
M	M	M	M	15	1	1	2	1	1	1	M	2	0	4	1	1	0	M	1	2
1	M	2	1	15	1	4	1	0	0	M	1	3	0	4	0	1	0	M	1	1
1	M	1	1	15	1	4	5	4	1	3	M	1	1	4	1	1	1	1	1	1
1	M	1	0	15	1	5	15	1	1	3	M	1	0	4	1	1	0	M	1	1
3	M	1	1	15	1	1	8	5	1	1	M	1	1	4	1	1	1	1	3	1
3	M	1	0	17	1	1	12	4	1	1	M	1	0	4	1	1	1	4	1	1
M	88	3	0	14	1	1	0	1	0	M	88	4	0	4	0	1	1	4	1	1
3	M	2	0	15	1	1	5	6	1	1	M	1	0	4	1	1	0	M	1	1
1	M	1	1	15	1	1	1	3	1	1	M	1	1	4	0	1	1	4	1	1
3	M	1	0	9	1	14	20	4	1	1	M	1	0	4	1	1	1	2	1	1
3	M	1	1	20	1	4	0	2	1	1	M	M	0	4	0	1	1	1	1	1
M	M	M	M	15	1	1	2	1	1	1	M	2	0	4	1	1	0	M	1	2
1	M	2	1	15	1	4	1	0	0	M	1	3	0	4	0	1	0	M	1	1
3	M	1	0	17	1	1	12	4	1	1	M	1	0	4	1	1	1	4	1	1
M	88	3	0	14	1	1	0	1	0	M	88	4	0	4	0	1	1	4	1	1
1	M	1	1	15	1	1	1	3	1	1	M	1	1	4	0	1	1	4	1	1
1	M	1	1	8	1	2	3	1	1	1	M	1	1	4	1	1	1	2	1	1
1	M	1	1	19	1	4	0	2	1	1	M	1	1	4	1	1	1	2	1	3
1	M	1	1	16	1	3	1	1	1	1	M	1	0	4	1	1	1	2	1	1
1	M	1	1	17	1	1	5	1	1	1	M	1	0	4	1	0	1	4	1	1

2	M	2	0	16	1	2	5	2	1	1	M	2	0	4	1	1	1	4	1	1
1	M	1	0	18	1	1	0	8	1	1	M	M	0	4	1	1	1	M	1	1
M	M	M	M	20	1	1	15	1	1	2	M	1	1	4	1	1	1	M	1	1
M	M	M	M	14	1	4	5	0	1	1	M	1	1	4	1	1	1	1	1	1
1	M	1	1	13	1	3	0	1	1	1	M	1	1	4	1	1	1	1	1	1
1	M	1	1	17	1	1	0	0	1	1	M	1	0	4	1	1	0	M	1	1
1	M	1	1	18	1	1	0	3	1	1	M	M	0	4	1	1	0	M	1	1
1	M	1	1	15	1	4	1	2	1	1	M	1	0	4	1	1	0	M	1	1
M	M	M	M	15	1	1	14	1	1	1	M	1	0	4	1	1	1	1	1	1
1	M	1	0	16	1	1	2	1	1	1	M	1	0	4	1	1	1	4	1	1
1	M	1	1	18	1	1	10	1	1	1	M	1	1	4	1	1	1	4	1	1
3	M	1	1	15	1	4	0	3	1	1	M	1	1	4	1	1	1	3	3	1
M	M	M	M	22	1	1	0	1	1	1	M	1	0	4	1	1	1	2	1	1
1	M	1	1	16	1	1	5	2	1	1	M	1	1	4	1	1	1	4	1	1
3	M	1	1	18	1	4	7	2	1	1	M	1	1	4	1	1	1	2	1	1
1	M	1	0	18	1	1	5	3	1	1	M	1	0	4	1	1	1	2	1	1
3	M	1	0	16	1	1	1	1	1	3	M	1	0	4	1	1	1	4	12	1
2	M	1	0	15	1	1	20	3	1	1	M	1	1	4	1	1	0	M	1	1
1	M	1	1	22	1	4	2	4	1	2	M	1	1	4	1	1	0	M	1	1
1	M	1	1	16	1	1	6	3	1	1	M	1	1	4	1	1	1	4	1	3
1	M	1	0	18	1	5	5	11	1	1	M	1	0	4	1	1	0	M	1	2
1	M	1	0	20	1	4	20	3	1	1	M	1	1	4	1	1	1	4	1	1
1	M	1	1	15	1	1	5	10	1	3	M	1	1	4	1	0	1	4	1	1
1	M	1	1	17	1	1	6	1	1	3	M	1	0	4	1	1	1	4	1	3
3	M	1	1	13	1	1	28	21	1	1	M	1	1	4	1	1	1	4	1	1
1	M	1	1	14	1	1	1	2	1	2	M	1	0	4	1	1	0	M	2	1
1	M	1	1	13	1	1	24	21	1	1	M	1	0	4	1	1	1	4	1	1
1	M	1	0	14	1	1	6	3	1	2	M	1	0	4	1	1	0	M	2	1
1	M	1	1	19	1	1	3	11	1	1	M	1	0	4	1	1	1	4	3	2
1	M	1	1	16	1	1	3	2	1	1	M	1	1	4	1	1	1	4	1	1
1	M	1	1	17	1	1	5	1	1	1	M	1	0	4	1	0	1	4	1	1
2	M	2	0	16	1	2	5	2	1	1	M	2	0	4	1	1	1	4	1	1
1	M	1	0	18	1	1	0	8	1	1	M	M	0	4	1	1	1	M	1	1
M	M	M	M	15	1	1	14	1	1	1	M	1	0	4	1	1	1	1	1	1
1	M	1	0	16	1	1	2	1	1	1	M	1	0	4	1	1	1	4	1	1
1	M	1	1	18	1	1	10	1	1	1	M	1	1	4	1	1	1	4	1	1
3	M	1	1	15	1	4	0	3	1	1	M	1	1	4	1	1	1	3	3	1
3	M	1	0	16	1	1	1	1	1	3	M	1	0	4	1	1	1	4	12	1
2	M	1	0	15	1	1	20	3	1	1	M	1	1	4	1	1	0	M	1	1
1	M	1	1	22	1	4	2	4	1	2	M	1	1	4	1	1	0	M	1	1
1	M	1	1	16	1	1	6	3	1	1	M	1	1	4	1	1	1	4	1	3
1	M	1	0	18	1	5	5	11	1	1	M	1	0	4	1	1	0	M	1	2
1	M	1	0	20	1	4	20	3	1	1	M	1	1	4	1	1	1	4	1	1
1	M	1	1	15	1	1	5	10	1	3	M	1	1	4	1	0	1	4	1	1

P43	P44	P45	P46	P47	P48	P49	P50	P51	P52	P53	P54	P55	P56	P57	P58	P59	P60	P61	P62	P63
1	2	0	1	3	1	5	2	1	0	M	0	M	0	M	1	1	0	1	12	6
3	1	0	1	2	1	1	2	1	0	M	0	M	1	2	1	M	0	1	12	4
3	2	0	1	3	1	4	2	1	1	1	0	M	1	3	1	M	0	1	25	25
3	3	0	1	3	1	4	2	1	0	M	0	M	0	M	1	M	0	1	30	88
1	2	0	1	3	1	2	2	1	0	M	0	M	0	M	1	M	0	1	8	88
1	1	1	1	3	1	1	2	2	0	M	0	M	0	M	1	M	0	1	1	3
1	2	0	1	4	1	6	1	1	0	M	0	M	1	1	1	M	0	1	30	2
2	2	1	0	3	1	5	2	1	0	M	0	M	0	M	1	M	0	1	56	88
1	3	0	0	1	1	2	1	1	0	M	0	M	0	M	1	M	0	1	20	7
2	2	1	1	4	1	5	1	1	0	M	0	M	0	M	1	M	0	1	15	88
1	1	0	0	5	0	M	2	1	0	M	1	0	0	M	0	2	0	1	20	7
2	3	0	1	3	1	1	2	1	0	M	0	M	0	M	1	M	0	1	6	25
1	3	0	0	1	1	1	1	1	0	M	1	1	0	M	1	M	0	1	30	2
2	2	0	0	1	1	1	2	1	0	M	0	M	0	M	1	M	0	1	30	88
1	3	88	0	3	0	M	1	1	0	M	0	M	0	M	1	M	0	1	20	88
1	2	0	1	3	1	5	2	1	0	M	0	M	0	M	1	1	0	1	12	6
3	1	0	1	2	1	1	2	1	0	M	0	M	1	2	1	M	0	1	12	4
1	1	1	1	3	1	1	2	2	0	M	0	M	0	M	1	M	0	1	1	3
1	3	0	0	1	1	2	1	1	0	M	0	M	0	M	1	M	0	1	20	7
2	2	1	1	4	1	5	1	1	0	M	0	M	0	M	1	M	0	1	15	88
1	2	0	1	1	1	2	1	1	0	M	0	M	0	M	0	2	0	1	15	3
2	3	0	1	3	0	M	2	1	0	M	0	M	0	M	0	4	0	1	35	25
2	3	1	1	1	1	2	2	1	0	M	0	M	0	M	1	M	0	1	15	3
1	2	0	1	3	1	2	2	1	0	M	0	M	0	M	1	M	0	1	25	4
3	2	0	1	3	1	5	2	1	0	M	0	M	0	M	1	M	0	1	20	4
2	2	1	1	1	1	2	1	1	0	M	0	M	0	M	0	2	0	1	15	2
1	3	0	1	1	1	2	1	1	0	M	0	M	0	M	1	M	0	1	20	4
1	2	1	1	1	1	4	2	0	0	M	0	M	0	M	1	M	0	1	15	2
1	2	0	1	2	1	5	2	1	0	M	0	M	0	M	1	M	0	1	20	4
1	3	0	1	1	1	1	1	1	0	M	0	M	0	M	1	M	0	1	30	20
1	2	0	1	4	1	1	2	1	0	M	0	M	0	M	1	M	0	1	30	25
2	3	1	0	1	1	4	1	1	0	M	0	M	0	M	1	M	0	1	30	20
1	2	0	1	3	1	2	2	0	0	M	0	M	0	M	1	M	0	1	56	4
1	1	0	1	3	1	2	2	1	0	M	0	M	0	M	0	4	0	1	20	2
1	3	0	1	3	1	3	1	1	0	M	0	M	0	M	1	M	0	1	30	25
1	2	0	1	3	1	2	2	1	0	M	0	M	0	M	1	M	0	1	25	4
3	2	0	1	3	1	5	2	1	0	M	0	M	0	M	1	M	0	1	20	4
1	3	0	1	1	1	2	1	1	0	M	0	M	0	M	1	M	0	1	20	4
1	2	1	1	1	1	4	2	0	0	M	0	M	0	M	1	M	0	1	15	2
2	3	1	0	1	1	4	1	1	0	M	0	M	0	M	1	M	0	1	30	20
1	2	0	1	3	1	2	2	0	0	M	0	M	0	M	1	M	0	1	56	4
1	3	0	1	3	1	3	1	1	0	M	0	M	0	M	1	M	0	1	30	25
2	3	0	0	3	0	M	1	2	0	M	0	M	0	M	1	M	0	1	15	4
1	2	0	0	1	1	2	2	1	1	2	0	M	1	2	0	4	0	1	25	3
1	2	0	1	3	1	2	2	1	0	M	0	M	0	M	1	M	0	1	8	88
1	2	0	1	1	1	1	2	1	0	M	0	M	0	M	1	M	0	1	12	3

1	2	1	1	3	1	2	1	1	0	M	0	M	0	M	1	M	0	1	20	4
1	1	0	1	3	1	2	2	1	0	M	0	M	0	M	0	4	0	1	20	2
1	3	0	1	3	1	5	2	1	0	M	0	M	1	M	0	4	0	1	35	2
1	2	0	0	1	1	88	2	1	0	M	0	M	0	M	1	M	0	1	20	2
2	1	0	0	3	1	2	2	1	1	2	0	M	1	2	0	1	0	1	12	2
2	3	1	0	1	0	M	1	2	0	M	0	M	0	M	1	M	0	1	8	88
1	2	1	1	1	1	2	1	1	0	M	0	M	0	M	1	M	0	1	24	25
1	2	1	1	1	1	5	2	1	0	M	0	M	0	M	1	M	0	1	20	25
1	3	0	1	1	0	M	1	1	0	M	0	M	0	M	1	M	0	1	20	3
1	3	0	1	1	1	1	2	1	0	M	0	M	0	M	0	1	0	1	25	6
1	3	0	1	1	1	2	2	1	0	M	0	M	0	M	1	M	0	1	12	20
2	3	0	0	3	0	M	2	1	0	M	0	M	0	M	1	M	0	1	20	3
3	2	0	1	3	1	4	1	1	0	M	0	M	0	M	1	M	0	1	25	3
1	3	0	1	3	1	4	2	1	0	M	0	M	0	M	1	M	1	1	25	0
1	1	0	0	1	1	1	2	1	0	M	1	0	1	2	1	M	0	1	20	88
1	3	0	1	4	1	2	1	1	0	M	0	M	0	M	0	4	0	1	20	4
1	3	0	1	1	0	M	1	1	0	M	0	M	0	M	1	M	0	1	20	20
1	3	0	0	3	0	M	1	1	0	M	0	M	0	M	1	M	0	1	15	2
1	3	0	1	1	1	2	2	0	0	M	1	88	0	M	1	M	0	1	30	2
2	2	0	0	2	1	1	2	1	0	M	0	M	0	M	1	M	0	1	30	7
1	2	0	1	1	1	2	2	1	0	M	0	M	0	M	88	1	0	1	30	20
1	3	0	1	1	0	M	2	1	0	M	0	M	0	M	1	M	0	1	30	20
1	3	0	1	1	0	M	2	1	0	M	0	M	0	M	1	M	0	1	20	7
2	3	0	0	1	0	M	1	1	0	M	0	M	0	M	1	M	0	1	30	20
1	3	0	1	1	1	1	1	1	0	M	0	M	1	1	1	M	0	1	25	88
2	3	0	1	1	1	4	1	1	0	M	0	M	0	M	1	M	0	1	20	88
2	2	0	1	1	1	1	2	1	0	M	0	M	0	M	1	M	0	1	20	88
2	2	0	1	1	1	1	1	1	0	M	0	M	0	M	1	M	0	1	20	2
2	3	0	0	3	0	M	1	1	0	M	0	M	0	M	1	M	0	1	20	88
1	2	0	1	2	0	M	2	1	0	M	0	M	0	M	1	M	0	1	30	25
1	2	0	1	1	1	1	2	1	0	M	0	M	0	M	1	M	0	1	12	3
1	2	1	1	3	1	2	1	1	0	M	0	M	0	M	1	M	0	1	20	4
1	1	0	1	3	1	2	2	1	0	M	0	M	0	M	0	4	0	1	20	2
1	3	0	1	1	0	M	1	1	0	M	0	M	0	M	1	M	0	1	20	3
1	3	0	1	1	1	1	2	1	0	M	0	M	0	M	0	1	0	1	25	6
1	3	0	1	1	1	2	2	1	0	M	0	M	0	M	1	M	0	1	12	20
2	3	0	0	3	0	M	2	1	0	M	0	M	0	M	1	M	0	1	20	3
1	3	0	1	1	0	M	1	1	0	M	0	M	0	M	1	M	0	1	20	20
1	3	0	0	3	0	M	1	1	0	M	0	M	0	M	1	M	0	1	15	2
1	3	0	1	1	1	2	2	0	0	M	1	88	0	M	1	M	0	1	30	2
2	2	0	0	2	1	1	2	1	0	M	0	M	0	M	1	M	0	1	30	7
1	2	0	1	1	1	2	2	1	0	M	0	M	0	M	88	1	0	1	30	20
1	3	0	1	1	0	M	2	1	0	M	0	M	0	M	1	M	0	1	30	20
1	3	0	1	1	0	M	2	1	0	M	0	M	0	M	1	M	0	1	20	7

P64	P65	P66	P67	P68	P69	P70	P71	P72	P73	P74	P75
0	1	0	1	1	1	1	1	0	1	0	88
1	0	1	1	8	1	1	1	0	1	0	0
0	1	0	1	1	1	1	1	1	1	1	1
0	0	1	2	M	M	M	1	1	1	0	0
1	1	1	1	1	0	1	1	0	1	0	0
1	0	0	1	7	1	0	1	1	1	0	0
0	0	0	1	9	1	1	1	0	0	0	0
0	0	0	2	M	M	M	1	0	1	0	0
0	0	1	2	M	M	M	1	1	1	1	0
0	0	0	2	M	M	M	1	1	1	0	0
0	0	0	2	M	M	M	1	1	1	1	1
0	0	1	2	M	M	M	1	0	1	88	0
0	0	0	2	M	M	M	1	1	1	0	0
0	0	1	2	M	M	M	1	1	1	0	0
0	0	1	2	M	M	M	1	1	0	88	1
0	1	0	1	1	1	1	1	0	1	0	88
1	0	1	1	8	1	1	1	0	1	0	0
1	0	0	1	7	1	0	1	1	1	0	0
0	0	1	2	M	M	M	1	1	1	1	0
0	0	0	2	M	M	M	1	1	1	0	0
1	0	0	0	M	1	1	1	0	1	0	0
0	0	0	2	M	M	M	1	1	1	0	1
0	1	0	0	M	1	1	1	1	1	0	1
0	0	0	2	M	M	M	1	1	1	0	0
0	0	0	2	M	M	M	1	1	1	0	0
0	0	0	2	M	M	M	1	1	1	88	88
0	0	0	2	M	M	M	1	1	1	1	0
0	0	0	2	M	M	M	1	0	1	0	1
0	1	1	0	M	M	M	1	1	1	0	0
0	0	1	2	M	M	M	1	1	1	1	1
0	1	1	1	1	0	1	1	1	1	0	0
0	0	0	2	M	M	M	1	0	1	0	0
1	0	0	2	M	M	M	1	0	1	88	1
0	0	0	2	M	M	M	1	0	1	88	0
0	0	1	2	M	M	M	1	1	1	1	0
0	0	0	2	M	M	M	1	1	1	0	0
0	0	0	2	M	M	M	1	1	1	0	0
0	0	0	2	M	M	M	1	1	1	1	0
0	0	0	2	M	M	M	1	0	1	0	1
0	0	0	2	M	M	M	1	0	1	0	0
1	0	0	2	M	M	M	1	0	1	88	1
0	0	1	2	M	M	M	1	1	1	1	0
0	0	0	2	M	M	M	1	1	1	1	0
0	0	1	2	M	M	M	1	1	1	0	0
88	88	0	0	M	M	M	1	0	1	0	0
0	0	0	2	M	M	M	1	0	1	0	0

0	0	1	2	M	M	M	1	1	1	88	0
0	0	0	2	M	M	M	1	0	1	0	0
0	0	1	2	M	M	M	1	1	1	0	0
0	0	0	2	M	M	M	1	1	1	0	0
1	0	0	1	1	1	1	1	0	1	1	0
0	0	0	2	M	M	M	1	1	1	0	0
0	0	0	2	M	M	M	1	0	1	0	1
0	0	0	2	M	M	M	1	1	1	0	0
0	0	0	2	M	M	M	1	0	1	1	0
0	0	0	2	M	M	M	1	0	1	88	88
0	0	0	2	M	M	M	1	1	1	0	88
0	0	0	2	M	M	M	1	1	1	0	0
1	0	0	1	1	1	1	1	0	1	0	0
0	1	0	0	M	M	M	1	1	1	0	0
1	0	0	2	M	M	M	1	0	1	1	0
0	0	1	2	M	M	M	1	1	1	0	1
0	0	0	2	M	M	M	1	0	0	0	0
0	0	0	2	M	M	M	1	0	1	0	1
0	0	1	2	M	M	M	1	1	1	1	0
0	0	1	2	M	M	M	1	1	1	0	1
0	0	1	2	M	M	M	1	1	1	0	0
0	0	1	1	8	1	1	1	1	1	0	0
0	0	0	2	M	M	M	1	0	1	0	0
0	0	1	2	M	M	M	1	1	1	1	0
0	0	1	2	M	M	M	1	0	1	1	0
0	0	0	2	M	M	M	1	1	1	0	0
0	0	1	2	M	M	M	1	0	1	0	88
0	0	0	2	M	M	M	1	1	1	1	0
0	0	0	2	M	M	M	1	1	1	0	0
0	0	1	2	M	M	M	1	0	1	0	0
0	0	0	2	M	M	M	1	0	1	0	0
0	0	1	2	M	M	M	1	1	1	88	0
0	0	0	2	M	M	M	1	0	1	0	0
0	0	0	2	M	M	M	1	0	1	1	0
0	0	0	2	M	M	M	1	0	1	88	88
0	0	0	2	M	M	M	1	1	1	0	88
0	0	0	2	M	M	M	1	1	1	0	0
0	0	0	2	M	M	M	1	0	0	0	0
0	0	0	2	M	M	M	1	0	1	0	1
0	0	1	2	M	M	M	1	1	1	1	0
0	0	1	2	M	M	M	1	1	1	0	1
0	0	1	2	M	M	M	1	1	1	0	0
0	0	1	1	8	1	1	1	1	1	0	0
0	0	0	2	M	M	M	1	0	1	0	0
